# Effects of Short- and Long-Term Vortioxetine Administration on Reproductive Function in Female Rats

**DOI:** 10.3390/ph18111690

**Published:** 2025-11-07

**Authors:** Eda Bingul, Elif Hizal, Ozgecan Keles, Nurinisa Yucel, Zeynep Suleyman, Murat Gunay, Hasan Yasar, Halis Suleyman

**Affiliations:** 1 Gynecology and Obstetrics Clinic, Erzurum City Hospital, 25240 Erzurum, Türkiye; 2 Gynecology and Obstetrics Clinic, Private Medicabil Hospital, 16350 Bursa, Türkiye; 3 Pharmacy Services Program, Vocational School of Health Services, Erzincan Binali Yildirim University, 24100 Erzincan, Türkiye; 4 Department of Internal Medicine Nursing, Faculty of Health Sciences, Erzincan Binali Yildirim University, 24030 Erzincan, Türkiye; 5 Medical Biochemistry Clinic, Mengucek Gazi Education and Research Hospital, 24100 Erzincan, Türkiye; 6 Department of Neurology, Faculty of Medicine, Erzincan Binali Yıldırım University, 24030 Erzincan, Türkiye; 7 Department of Pharmacology, Faculty of Medicine, Erzincan Binali Yildirim University, 24030 Erzincan, Türkiye

**Keywords:** corticosterone, infertility, oxidative stress, reproductive function, vortioxetine

## Abstract

**Background/Objectives**: Vortioxetine is a multimodal antidepressant that modulates serotonergic, noradrenergic, and dopaminergic systems, yet its effects on female reproductive physiology remain incompletely defined. This study investigated how short- and long-term vortioxetine exposure influences reproductive function in female rats, integrating measurements of oxidative-stress biomarkers, catecholamines, and endocrine endpoints (prolactin and corticosterone). **Methods**: Forty-two female Wistar albino rats were randomized into seven groups (*n* = 6): healthy control; short-term vortioxetine; long-term vortioxetine; and the same two regimens each combined with metyrosine or metyrapone. Metyrosine and metyrapone (50 mg/kg, oral gavage) were given 1 h before vortioxetine (10 mg/kg). Treatments lasted one week (short-term) or four weeks (long-term). Serum MDA, tGSH, SOD, CAT, adrenaline, noradrenaline, dopamine, serotonin, prolactin, and corticosterone were measured. Fertility outcomes were assessed by co-housing females with males for one month. **Results**: Vortioxetine increased MDA, catecholamines, and serotonin and decreased antioxidant markers and prolactin versus controls (*p* < 0.001). Long-term vortioxetine produced a marked rise in corticosterone that coincided with infertility and delayed parturition. Metyrosine lowered oxidative stress and catecholamines but did not reverse infertility. In contrast, metyrapone blunted corticosterone elevation and preserved reproductive capacity. **Conclusions**: Long-term vortioxetine induced infertility in female rats, likely mediated by corticosterone elevation consistent with hypothalamic–pituitary–adrenal axis dysregulation. These findings suggest the need to monitor reproductive risk when considering vortioxetine in women of reproductive age and warrant further mechanistic and translational studies.

## 1. Introduction

Vortioxetine (1-[2-[2-(2,4-dimethyl-phenylsulfonyl-phenyl) phenyl] phenyl]-piperazine hydrobromide) is a multimodal antidepressant approved for major depressive disorder [1]. Vortioxetine’s pharmacological profile is characterized by inhibition of the serotonin transporter (SERT), agonism at 5-hydroxytryptamine (5-HT)1A receptors, partial agonism at 5-HT_1_B receptors, and antagonism at 5-HT_3_, 5-HT_7_, and 5-HT_1_D receptors [1,2,3]. Notably, vortioxetine has been reported to produce nearly twice the extracellular serotonin levels observed with selective serotonin reuptake inhibitors (SSRIs), and preclinical data indicate that 5-HT1A agonism can accelerate desensitization of 5-HT1A autoreceptors, thereby enhancing serotonergic neurotransmission [4].

In addition to serotonergic actions, vortioxetine modulates noradrenergic, dopaminergic, cholinergic, histaminergic, glutamatergic, and GABAergic neurotransmission [1]. Activation of 5-HT1A receptors has been linked to increased release of ACTH, cortisol, and catecholamines, as well as elevations in β-endorphins, oxytocin, and prolactin [5]. In patients with glucocorticoid resistance, vortioxetine has been suggested to improve stress responsiveness [6]. Activation of 5-HT_1_A receptors modulates ACTH and corticosterone release, potentially linking vortioxetine to hypothalamic–pituitary–adrenal (HPA) axis activation and reproductive hormone imbalance [5].

The role of serotonin in reproductive physiology, however, remains contested. Elevated circulating serotonin has been associated with male infertility [7], and serotonin can increase prolactin and cortisol via multiple mechanisms [8,9]; heightened levels of these hormones have been implicated in menstrual irregularities and infertility [10]. Moreover, the use of serotonin reuptake inhibitors has been associated with reduced efficacy of infertility treatments, increased pregnancy loss, preterm delivery, and long-term neurobehavioral abnormalities in offspring [11].

Evidence regarding vortioxetine’s redox effects is likewise mixed: while some studies report induction of oxidative-stress responses, others suggest suppression via activation of antioxidant defenses in certain models [12,13]. Critically, no systematic investigation has simultaneously assessed vortioxetine’s effects on oxidative-stress biomarkers together with serum prolactin and corticosterone (the principal rodent glucocorticoid, analogous to cortisol) levels, and data on its impact on female reproductive function remain limited.

Accordingly, this study evaluated the effects of short- and long-term vortioxetine administration on reproductive function in female rats. We quantified oxidant/antioxidant parameters—malondialdehyde (MDA), total glutathione (tGSH), superoxide dismutase (SOD), and catalase (CAT)—alongside catecholamines [adrenaline (ADR), noradrenaline (NDR), dopamine (DOP)], serotonin, prolactin, and corticosterone, to delineate potential endocrine and redox mechanisms underlying vortioxetine-related reproductive dysfunction.

## 2. Results

### 2.1. Serum MDA, tGSH, SOD and CAT Analysis Results

**Figure 1:**
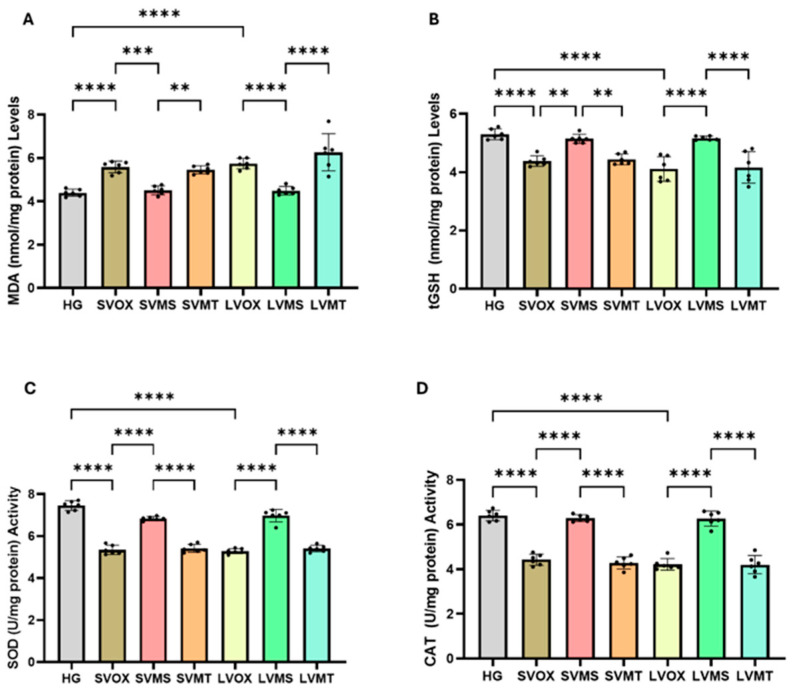
Effects of vortioxetine and co-treatments on serum oxidative status (normalized to protein). (**A**) MDA (nmol/mg protein), (**B**) tGSH (nmol/mg protein), (**C**) SOD (U/mg protein), and (**D**) CAT (U/mg protein). Bars indicate mean ± standard deviation (SD); *n* = 6 per group. Black dots represent individual animal values. Statistical comparisons between groups were performed using one-way ANOVA followed by Tukey’s post hoc test. Statistical significance was set at *p* < 0.05. Significance levels: ** *p* < 0.01, *** *p* < 0.001, **** *p* < 0.0001. Abbreviations: HG, healthy; SVOX, short-term VOX; SVMS, short-term VOX + metyrosine; SVMT, short-term VOX + metyrapone; LVOX, long-term VOX; LVMS, long-term VOX + metyrosine; LVMT, long-term VOX + metyrapone.

As shown in Figure 1A and Table 1, serum MDA levels were significantly elevated in both the short-term (SVOX) and long-term (LVOX) vortioxetine groups compared with the healthy control (HG) (*p* < 0.0001). Conversely, levels of tGSH, SOD, and CAT were significantly reduced in these groups (*p* < 0.0001 for all) (respectively, Figure 1B–D and Table 1). Coadministration of metyrosine (SVMS and LVMS) significantly attenuated the vortioxetine-induced increase in MDA (*p* = 0.0004 and <0.0001, respectively) and effectively preserved tGSH (*p* = 0.0012 and <0.0001), SOD (both *p* < 0.0001), and CAT (both *p* < 0.0001) levels. In contrast, metyrapone (SVMT and LVMT) showed only partial or no protective effect: although MDA levels were mildly reduced in SVMT vs. SVOX (*p* = 0.0023), this was not observed in LVMT (*p* = 0.996 vs. LVOX). Similarly, tGSH, SOD, and CAT levels in LVMT remained significantly lower than in HG and were not different from LVOX (all *p* > 0.95). One-way ANOVA confirmed significant group differences in all markers: MDA (F(6,35) = 22.542), tGSH (F(6,35) = 18.424), SOD (F(6,35) = 136.738), and CAT (F(6,35) = 88.728); *p* < 0.0001 for all. There were no significant differences between the short- and long-term metyrosine groups (SVMS vs. LVMS) for MDA (*p* > 0.9999), tGSH (*p* > 0.9999), SOD (*p* = 0.884), or CAT (*p* > 0.9999).

### 2.2. Serum ADR, NDR, DOP and Serotonin Analysis Results

**Figure 2:**
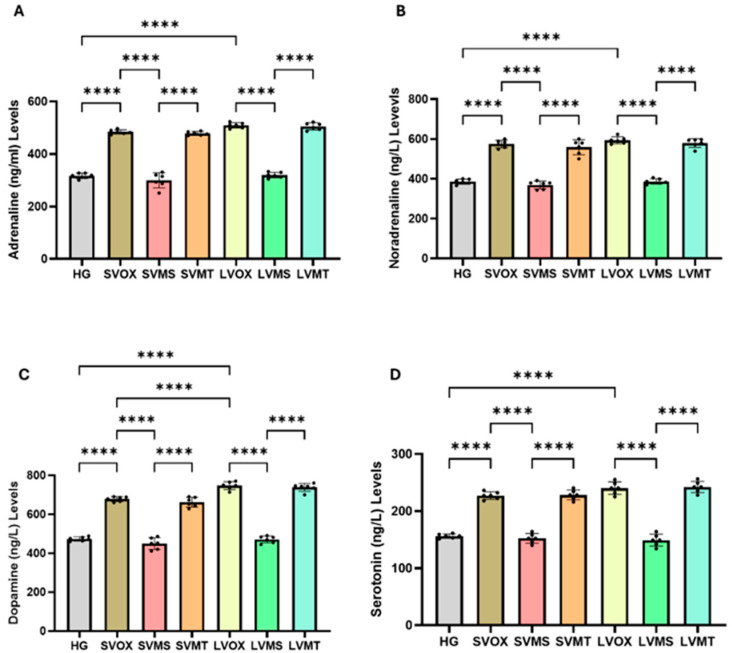
Effects of vortioxetine and co-treatments on serum catecholamine and serotonin levels. (**A**) Adrenaline (ADR, ng/mL), (**B**) Noradrenaline (NDR, ng/mL), (**C**) Dopamine (DOP, ng/mL), and (**D**) Serotonin (ng/mL). Bars indicate mean ± standard deviation (SD); *n* = 6 per group. Black dots represent individual animal values. Statistical comparisons between groups were performed using one-way ANOVA followed by Tukey’s post hoc test. Statistical significance was set at *p* < 0.05. Significance levels **** *p* < 0.0001. Abbreviations: HG, healthy; SVOX, short-term VOX; SVMS, short-term VOX + metyrosine; SVMT, short-term VOX + metyrapone; LVOX, long-term VOX; LVMS, long-term VOX + metyrosine; LVMT, long-term VOX + metyrapone.

As shown in Figure 2 and Table 2, both short- and long-term administration of vortioxetine led to a significant increase in serum ADR, NDR, DOP and serotonin levels compared with the healthy control group (HG) (*p*  <  0.0001 for all comparisons). Among the vortioxetine-treated groups, dopamine levels were significantly higher in the long-term vortioxetine group (LVOX) compared to the short-term vortioxetine group (SVOX) (*p*  <  0.0001). However, no significant differences were observed between these two groups for adrenaline (*p*  =  0.0686), noradrenaline (*p*  =  0.7633), or serotonin levels (*p*  =  0.1556). Coadministration of metyrosine significantly suppressed the vortioxetine-induced elevations in ADR, NDR, DOP, and serotonin under both short- and long-term treatment conditions (*p*  <  0.0001 for all parameters). In contrast, metyrapone coadministration failed to significantly alter the elevations caused by vortioxetine, with no significant differences compared to the vortioxetine-only groups (all *p*  >  0.05). Furthermore, no significant differences were observed between SVMS and LVMS groups for ADR (*p* = 0.2243), NDR (*p* = 0.7853), DOP (*p* = 0.5616), or serotonin (*p* = 0.9964). One-way ANOVA confirmed significant group differences in all markers: ADR (F(6,35) = 275.6), NDR (F(6,35) = 141.4), DOP (F(6,35) = 253.3), and serotonin (F(6,35) = 156.7); *p* < 0.0001 for all.

### 2.3. Serum Prolactin Analysis Results

**Figure 3:**
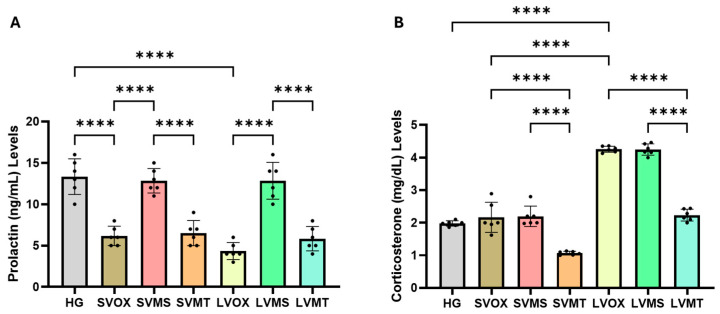
Effects of vortioxetine and co-treatments on serum prolactin (**A**) and corticosterone (**B**) levels (mg/dL). Bars indicate mean ± standard deviation (SD); *n* = 6 per group. Black dots represent in-dividual animal values. Statistical comparisons between groups were performed using one-way ANOVA followed by Tukey’s post hoc test. Statistical significance was set at *p* < 0.05. Significance levels **** *p* < 0.0001. Abbreviations: HG, healthy; SVOX, short-term VOX; SVMS, short-term VOX + metyrosine; SVMT, short-term VOX + metyrapone; LVOX, long-term VOX; LVMS, long-term VOX + metyrosine; LVMT, long-term VOX + metyrapone.

As shown in Figure 3A and Table 2 short-term vortioxetine administration (SVOX) led to a significant reduction in prolactin levels compared to the healthy group (HG) (*p* < 0.0001), and this suppression was even more pronounced in the long-term vortioxetine group (LVOX) (*p* < 0.0001). Coadministration of Metyrosine (SVMS and LVMS) significantly reversed the vortioxetine-induced reduction in prolactin levels in both short- and long-term treatment groups (*p* < 0.0001 vs. SVOX and LVOX, respectively). However, there were no significant differences between SVMS and LVMS groups (*p* > 0.9999), indicating that the prolactin-restorative effect of metyrosine was consistent over time. Conversely, metyrapone co-treatment (SVMT and LVMT) did not significantly affect the suppression of prolactin by vortioxetine (all *p* > 0.05), indicating a lack of counteractive potential on this parameter. One-way ANOVA confirmed significant group differences in serum prolactin levels (F(6,35) = 35.21, *p* < 0.0001).

### 2.4. Serum Corticosterone Analysis Results

As shown in Figure 3B and Table 2, short-term administration of vortioxetine (SVOX) did not significantly alter serum corticosterone levels compared with the healthy control group (HG) (*p* = 0.8086). However, long-term vortioxetine administration (LVOX) resulted in a marked and statistically significant increase in corticosterone levels compared with both the HG and SVOX groups (*p* < 0.0001 for both comparisons). Metyrapone co-administration (LVMT) effectively reversed the vortioxetine-induced increase in corticosterone levels (*p* < 0.0001 vs. LVOX), bringing the levels close to baseline. In contrast, metyrosine (LVMS) failed to mitigate this effect, as corticosterone levels remained significantly elevated compared to the HG and SVOX groups (*p* < 0.0001). A strong positive correlation was found between mean serum corticosterone levels and infertility outcome (r = 0.93, *p* < 0.01; Supplementary Figure S1).

### 2.5. Reproductive Test Analysis Results

As shown in Table 3, no infertility was observed in the healthy group or in animals treated with short-term vortioxetine (SVOX, SVMS, SVMT). These groups demonstrated elevated catecholamine levels and reduced prolactin, whereas corticosterone remained largely unaffected, particularly in metyrapone-treated animals. By contrast, reproductive dysfunction was evident in the groups receiving long-term vortioxetine (LVOX, LVMS). In these animals, oxidative damage and catecholamine elevation were accompanied by a more pronounced reduction in prolactin and, importantly, a marked increase in corticosterone. This excessive rise in corticosterone was strongly associated with infertility: 4 out of 6 rats in the LVOX group and 3 out of 6 rats in the LVMS group were infertile.

None of the rats in the LVMT group, which received metyrapone in combination with vortioxetine, developed infertility. Thus, metyrapone co-treatment preserved fertility, although it did not prevent a prolonged maternity period. The mean interval from mating to delivery was 24.5, 24.5, 24.7, 23.3, 32.5, 33.0, and 23.7 days in the HG, SVOX, SVMS, SVMT, LVOX, LVMS, and LVMT groups, respectively. The corresponding delays in the maternity period were 2.5, 2.5, 2.7, 2.5, 1.3, 10.5, and 11.0 days (Table 2). A strong positive correlation was found between mean serum corticosterone levels and infertility outcome (r = 0.93, *p* < 0.01; Supplementary Figure S1).

## 3. Discussion

In the present study, short-term vortioxetine administration did not result in reproductive dysfunction in female rats, whereas long-term exposure caused both infertility and delayed parturition. Moreover, long-term treatment was associated with significantly elevated corticosterone levels, which demonstrated a positive correlation with infertility. The primary aim of this study was to investigate the reproductive effects of vortioxetine in female rats following short- and long-term exposure, while also exploring the potential contributions of oxidative stress, catecholamines, prolactin, and corticosterone to the underlying pathophysiology.

Our findings indicate that vortioxetine induces alterations in oxidative/antioxidant balance, catecholamine turnover, and endocrine parameters, all of which are linked to reproductive outcomes. Previous reports have described contradictory effects of vortioxetine, demonstrating both pro-oxidant and antioxidant properties [12,13]. In this study, oxidative stress was evidenced by increased MDA levels alongside decreased tGSH, SOD, and CAT activity in all vortioxetine-treated groups, consistent with established oxidative markers [14,15].

Catecholamine levels (ADR, NDR, DOP) were significantly elevated in vortioxetine-treated groups, where oxidative damage was high and antioxidant capacity low. This is consistent with prior studies showing that vortioxetine increases catecholamines [1,5], and that catecholamine elevations are positively correlated with oxidative stress and inversely related to antioxidant status [16,17,18]. Metyrosine effectively suppressed both catecholamine elevation and oxidative imbalance, in line with its tyrosine hydroxylase–inhibitory and antioxidant properties [19,20,21]. In contrast, metyrapone—a cortisol synthesis inhibitor [22]—did not alter the oxidative or catecholaminergic changes induced by vortioxetine, supporting the concept that oxidative stress is primarily driven by catecholamine excess rather than corticosterone dysregulation.

Given the reported negative impact of SSRIs on ovulation and female reproductive capacity [23], serum serotonin levels were also evaluated. Vortioxetine increased serotonin following both short- and long-term administration; this effect was prevented by metyrosine. However, normalization of serotonin did not reverse infertility in long-term vortioxetine groups, suggesting that reproductive dysfunction develops independently of serotonin.

Our findings support a multi-hit stress model rather than a single-parameter explanation. Long-term vortioxetine exposure was associated not only with elevated corticosterone but also with increased catecholaminergic tone and oxidative stress. Sustained catecholamine release is known to drive mitochondrial reactive oxygen species generation and lipid peroxidation, thereby imposing persistent oxidative load on central and peripheral tissues [19,20,21]. Chronic redox imbalance can, in turn, destabilize glucocorticoid feedback and maintain a hyperactivated hypothalamic–pituitary–adrenal (HPA) axis, resulting in prolonged corticosterone elevation [24,25]. Glucocorticoid excess and corticotropin-releasing hormone signaling are well recognized to suppress hypothalamic gonadotropin-releasing hormone (GnRH) pulsatility, blunt luteinizing hormone (LH) and follicle-stimulating hormone (FSH) output, and impair follicular maturation and ovulation, ultimately reducing fertility [26]. In our study, metyrosine—by limiting catecholamine synthesis and improving oxidative indices [19,20,21]—partially corrected the redox environment but did not normalize fertility, whereas metyrapone, which inhibits corticosterone synthesis [22], preserved fertility despite ongoing serotonergic modulation. These results suggest that catecholamine-driven oxidative stress and HPA-axis hyperactivity act in concert, with sustained corticosterone signaling emerging as a necessary downstream effector of infertility in chronically treated animals.

Prolactin, a key hormone in female reproduction [10], was reduced by vortioxetine in both short- and long-term treatment groups. While prolactin levels normalized with metyrosine co-treatment, they remained unchanged with metyrapone. This finding is in contrast to most previous reports, which associate serotonin elevation with hyperprolactinemia [27,28,29]. Elevated dopamine, observed in this study, may have exerted an inhibitory effect on prolactin secretion through D2 receptor stimulation [30,31]. The recovery of prolactin levels following metyrosine treatment further supports this mechanism.

Vortioxetine, as a serotonergic drug, also influences the hypothalamic–pituitary–adrenal (HPA) axis [32]. Although short-term treatment did not alter corticosterone, long-term vortioxetine significantly increased corticosterone, consistent with elevated serotonergic activity [33,34]. Interestingly, long-term vortioxetine combined with metyrosine reduced serotonin but did not normalize corticosterone, suggesting that factors other than serotonin contribute to HPA axis activation. Metyrapone effectively suppressed the vortioxetine-induced corticosterone increase, aligning with its known inhibitory action on 11β-hydroxylase [35]. Previous clinical studies have shown that SSRIs elevate cortisol in humans [36,37], which is consistent with our data, although one study reported decreased corticosterone in female rats following vortioxetine [38]. The inclusion of metyrosine and metyrapone provided mechanistic insight into vortioxetine-induced reproductive dysfunction. Metyrosine inhibits catecholamine synthesis and alleviates oxidative stress [19,20,21], whereas metyrapone blocks corticosterone synthesis by inhibiting 11β-hydroxylase [22]. The observation that metyrapone, but not metyrosine, preserved fertility supports the hypothesis that chronic HPA-axis activation and hypercorticosteronemia—rather than catecholamine excess—underlie the observed infertility.

In the reproductive analysis, infertility was observed exclusively in long-term vortioxetine groups with elevated corticosterone. High cortisol levels are known to impair pituitary sensitivity to GnRH and LH, thereby disrupting ovulation [10]. Human studies also support a negative association between cortisol and fertility in women undergoing IVF [39]. Casillas et al. similarly demonstrated that chronic stress–induced hypercortisolemia correlates with infertility and prolonged gestation in female rats [40,41]. Although our study did not evaluate estrous cycle phases, which can influence serotonergic activity [42], this limitation should be considered when interpreting the data. However, clinical evidence regarding endocrine and reproductive hormonal effects of vortioxetine in women remains scarce, and further studies are warranted to clarify its long-term impact on female endocrine function.

Interestingly, while long-term vortioxetine decreased prolactin, infertility was more closely associated with corticosterone elevation rather than prolactin suppression. Metyrapone preserved fertility despite low prolactin, whereas metyrosine failed to prevent infertility despite normalizing prolactin levels. These results indicate that prolactin is not the primary mediator of vortioxetine-related infertility. Literature on hypoprolactinemia and reproductive behavior remains inconsistent: while some studies link low prolactin to reduced sexual drive in humans, others report minimal or no impact on fertility in rodents [43,44].

Consistent with previous findings by Cim et al. [45], our study confirmed that vortioxetine induces oxidative stress. Importantly, this effect occurred even in unstressed animals, suggesting a direct pro-oxidant effect of vortioxetine. Taken together, our data demonstrates that long-term vortioxetine exposure induces oxidative stress, elevates catecholamine and corticosterone levels, decreases prolactin, and ultimately leads to infertility.

### Limitations

Future studies should include histopathological and immunohistochemical evaluation of ovarian tissue, as well as tissue-level assessment of oxidative stress markers. Monitoring of the estrous cycle and sexual behavior (e.g., vaginal plate monitoring) would further clarify the reproductive impact of vortioxetine. Although infertility closely paralleled corticosterone elevation, this association does not prove causation. Definitive mechanistic evidence would require corticosterone replacement or blockade experiments. The fertility-preserving effect of metyrapone supports—but does not conclusively demonstrate—a causal role of glucocorticoid excess in vortioxetine-induced infertility. In addition to excess corticosterone, disrupted gonadotropin (LH/FSH) or estradiol signaling may underlie reduced fertility. Chronic serotonergic stimulation has been reported to suppress GnRH and lower gonadotropin release [11,39,40]; thus, concurrent endocrine alterations cannot be excluded and warrant investigation in future studies. The absence of vehicle + metyrosine and vehicle + metyrapone groups represents a limitation of the current design, preventing full assessment of potential drug–drug interactions. Future studies will incorporate these control groups to strengthen causal interpretation.

## 4. Materials and Methods

### 4.1. Animals

Animals were grouped according to sex and body weight, and those exhibiting decreased weight gain, hunched posture, aggressive behavior, piloerection, or reduced food and water intake were excluded from the study. A total of forty-two female Wistar albino rats (248–255 g, 5–6 months old) were used. All animals were obtained from the Medical Experimental Research and Application Centre of Binali Yildirim University. Rats were housed under controlled environmental conditions at 22 °C, with free access to standard laboratory chow and water, and maintained on a 12 h light/12 h dark cycle prior to the experiment. Six animals were housed per cage. The sample size was determined based on previous literature and the 4R principle.

All experimental procedures were approved by the Erzincan Binali Yildirim University Local Animal Experimentation Ethics Committee (approval code 2024/03/344733 and approved on 28 March 2024, Meeting No: 15) and conducted in compliance with EU Directive 2010/63/EU for the care and use of laboratory animals and the ARRIVE guidelines.

### 4.2. Chemical Substances

Ketamine was obtained from Pfizer Pharmaceuticals Ltd. (Istanbul, Türkiye), and vortioxetine from Lundbeck Pharmaceuticals Ltd. (Istanbul, Türkiye). Metyrapone was purchased from Alliance Pharmaceutical Ltd. (Chippenham, UK), and metyrosine from Sigma Chemical Co., Ltd. (Munich, Germany).

### 4.3. Experimental Groups

The rats utilized in the study were separated into seven groups as follows; healthy (HG), short-term vortioxetine alone (SVOX), short-term vortioxetine + Metyrosine (SVMS), short-term vortioxetine + Methyrapone (SVMT), long-term vortioxetine alone (LVOX), long-term vortioxetine + Metyrosine (LVMS), long-term vortioxetine + Methyrapone (LVMT).

### 4.4. Experimental Procedure

To initiate the experiment, metyrosine (50 mg/kg, twice daily) was administered orally by gavage to the SVMS group (*n* = 6), and metyrapone (50 mg/kg, once daily) was administered by gavage to the SVMT group (*n* = 6). Metyrosine, a tyrosine hydroxylase inhibitor, reduces the synthesis of adrenaline and noradrenaline [46] and has also been reported to possess antioxidant activity [47]. Metyrapone suppresses adrenal cortisol production through inhibition of the 11β-hydroxylase enzyme [48]. These drugs were used to investigate the effects of vortioxetine on catecholamines and hormones when their levels were pharmacologically reduced by metyrosine and metyrapone. The SVOX (*n* = 6) and HG (*n* = 6) groups received physiological saline as vehicle. One hour after drug or saline administration, the SVOX, SVMS, and SVMT groups were treated with vortioxetine (10 mg/kg, once daily) by the same method. This protocol was continued for one week [49,50,51]. At the end of this period, blood samples were collected from the tail veins, and the animals were housed with males for one month to assess reproductive performance. Serum oxidant, antioxidant, ADR, NDR, DOP, serotonin, prolactin, and corticosterone levels were analyzed.

For the long-term experiment, the same procedure was applied to the LVMS (*n* = 6) and LVMT (*n* = 6) groups, which received metyrosine (50 mg/kg, twice daily) or metyrapone (50 mg/kg, once daily), respectively. Physiological saline was given to the HG and LVOX groups (*n* = 6). One hour later, vortioxetine (10 mg/kg, once daily) was administered to the LVOX, LVMS, and LVMT groups by gavage for four weeks [52]. This regimen yields plasma levels analogous to those observed in patients treated with 10–20 mg/day vortioxetine [3,4], supporting translational comparability. At the end of treatment, blood samples were obtained from the tail veins to measure oxidants, antioxidants, catecholamines, prolactin, and corticosterone. Following the treatment period, each female rat was cohabited with a proven fertile, untreated male for one month. Pregnancy status was evaluated through physical examination and abdominal palpation. Pregnant rats were subsequently housed individually under controlled environmental conditions until parturition, while those that did not deliver within one month were classified as infertile. Fertility rate (%) was calculated as (number of pregnant females/total number of females) × 100, as shown in Table 2.

All animals were euthanized with a high dose of ketamine (150 mg/kg) [53]. Biochemical and hormonal analyses were conducted in a single-blind manner by investigators who were unaware of the group assignments.

### 4.5. Measurement of MDA, tGSH, SOD, and CAT Determination in the Serum

All analyses were performed on supernatants obtained from serum samples using enzyme-linked immunosorbent assay (ELISA). Commercial ELISA kits were used for the quantification of MDA, tGSH, and SOD levels, following the manufacturer’s instructions (Cat. No: 10009055, 706002, and 703002; Cayman Chemical Company, Ann Arbor, MI, USA). CAT activity was determined according to the method described by Goth [54]. Protein concentration was measured spectrophotometrically at 595 nm using the Bradford assay [55].

### 4.6. Measurement of ADR, NDR, DOP and Serotonin Levels in the Serum

To determine ADR, NDR, and DOP levels, blood samples were collected from the serum. The ethylenediamine tetra-acetic acid (EDTA) samples were taken out ice and centrifuged at 3500 rpm for 15 min and were frozen and stored at −80 °C. After centrifugation, plasma ADR, NDR and DOP concentrations were analyzed by a high-performance liquid chromatography (HPLC) pump and an isocratic system using an electrochemical detector. For HPLC (Hewlett Packard Agilent 1100; Hewlett Packard Enterprise, Spring, TX, USA; flow rate: 1 mL/min; injection volume: 40 μL; analytical run time: 20 min) analysis of plasma-serum catecholamines, we used a reagent kit (Chromsystems, Munich, Germany). Quantitative determination of serotonin molecule (5-Hydroxytryptamine) was performed using LC-MS/MS (Liquid chromotography Mass Spectrometry) method and LC-MS/MS Analysis Kit (Sem Laboratory Devices Marketing Inc., Istanbul, Türkiye).

### 4.7. Prolactin and Corticosterone Quantifications in the Serum

Blood samples were centrifuged at 1200× *g* and serum samples obtained from the supernatant were measured for prolactin levels using an immunoassay analyzer (Abbott Architect immunoassay, Abbott Laboratories, Chicago, IL, USA) and Architect Prolactin kit (Cat No: 7K7625, Abbott, Wiesbaden, Germany). Analysis was carried out following kit instructions. All samples were centrifuged at 3500× *g* for 10 min and were frozen and stored at −80 °C. The plasma was filtered with 5 mL of ethyl acetate. It was then cleaned with sodium hydroxide (0.1 M) and water. After evaporating the ethyl acetate, the residue was dissolved in the mobile phase (acetonitrile-water-acetic acid-triethanolamine 22:78:0.1:0.03, *v*/*v*) and injected into an isocratic high-performance liquid chromatography (HPLC) system. The concentration of cortisol in the plasma was measured by isocratic system with an HPLC pump (Model Hewlett Packard Agilent 1100, Agilent Technologies, Santa Clara, CA, USA). Purified corticosterone (Sigma, St. Louis, MO, USA) was prepared, dissolved in ethyl acetate, and checked against normal corticosterone.

### 4.8. Statistical Analysis

In this experimental study IBM SPSS 22^®^ (IBM Corp., Armonk, NY, USA) program was used for all analyses. Graphs created in GraphPad Prism 10.5.0. The normality assumption of the biochemical parameters was evaluated using the Kolmogorov–Smirnov test, and the homogeneity of variances assumption was checked using Levene’s test. Differences between groups were obtained using one-way analysis of variance (ANOVA) for normally distributed variables. Tukey’s honestly significant difference (HSD) test or Games-Howell test was applied as a post hoc test, according to whether or not the normality of variances assumption was met. Pearson Correlation Analysis was performed to examine the relationship between corticosterone and infertility. Results were expressed as “mean ± standard deviation” (X ± SD). Statistic meaning was set at *p* < 0.05.

## 5. Conclusions

Short-term vortioxetine use did not impair reproductive function, whereas long-term treatment caused infertility and delayed maternity. Taken together, the findings suggest that long-term vortioxetine exposure may impair fertility through corticosterone-mediated mechanisms, while short-term treatment appears safe in this regard. In clinical settings, these data support monitoring of menstrual function and, when necessary, evaluation of prolactin and cortisol levels during prolonged therapy, particularly in women of reproductive age or those undergoing infertility treatment.

## Figures and Tables

**Table 1:** Analysis results of oxidative stress and antioxidant parameters obtained from experimental groups.

Biochemical Variables	Experimental Groups (*n* = 6/Each Group)						
	HG	SVOX	SVMS	SVMT	LVOX	LVMS	LVMT
	Mean ± Standard Deviation						
MDA (nmol/mg protein)	4.39 ± 0.16 #‡	5.58 ± 0.27 *	4.51 ± 0.20 #‡	5.45 ± 0.19 *	5.73 ± 0.24 †	4.49 ± 0.19 #‡	6.26 ± 0.86 †
tGSH (nmol/mg protein)	5.30 ± 0.18 #‡	4.38 ± 0.18 *	5.15 ± 0.16 #‡	4.44 ± 0.18 *	4.11 ± 0.43 †	5.17 ± 0.07 #‡	4.16 ± 0.54 †
SOD (U/mg protein)	7.45 ± 0.23 #‡	5.35 ± 0.22 *	6.84 ± 0.09 *#‡	5.42 ± 0.19 *	5.29 ± 0.12 †	6.98 ± 0.30 †#‡	5.41 ± 0.16 †
CAT (U/mg protein)	6.41 ± 0.24 #‡	4.43 ± 0.25 *	6.29 ± 0.15 #‡	4.29 ± 0.27 *	4.23 ± 0.26 †	6.27 ± 0.33 #‡	4.21 ± 0.41 †

The results are presented as mean ± standard deviation. For all groups *n* = 6. Symbols (Tukey, *p* < 0.05): * vs. HG (short-term groups: SVOX, SVMS, SVMT); † vs. HG (long-term groups: LVOX, LVMS, LVMT); # vs. SVOX (all other groups compared with SVOX); ‡ vs. LVOX (all other groups compared with LVOXAbbreviations: MDA; malondialdehyde, tGSH; total glutathione, SOD; superoxide dismutase, CAT; catalase, HG; healthy, SVOX; short-term vortioxetine alone, SVMS; short-term vortioxetine + Metyrosine, SVMT; short-term vortioxetine + Methyrapone, LVOX; long-term vortioxetine alone, LVMS; long-term vortioxetine + Metyrosine, LVMT; long-term vortioxetine + Methyrapone.

**Table 2:** Analysis results of hormonal and neurotransmitter parameters obtained from experimental groups.

Biochemical Variables	Experimental Groups (*n* = 6/Each Group)						
	HG	SVOX	SVMS	SVMT	LVOX	LVMS	LVMT
	Mean ± Standard Deviation						
Adrenalin (ng/mL)	317.00± 10.30 #‡	484.17 ± 8.42 *	299.50 ± 28.99 #‡	478.50 ± 7.15 *‡	509.17 ± 10.26 †	319.67 ± 10.50 #‡	505.17 ± 13.59 †
Noradrenaline (ng/L)	385.83 ± 12.81 #‡	574.83 ± 19.18 *	369.00 ± 19.69 #‡	558.17 ± 37.54 *	593.17 ± 17.52 †	386.83 ± 13.01 #‡	579.17 ± 22.09 †
Dopamine (ng/L)	473.33 ± 10.65 #‡	678.00 ± 12.35 *‡	449.50 ± 30.01 #‡	662.17 ± 25.09 *‡	747.17 ± 20.36 †#	470.67 ± 17.53 #‡	737.83 ± 20.25 †#
Serotonin (ng/L)	156.00 ± 3.69 #‡	227.00 ± 6.51 *	152.00 ± 8.49 #‡	228.00 ± 8.49 *	240.00 ± 10.75 †	149.00 ± 10.47 #‡	242.00 ± 9.90 †
Prolactin (ng/mL)	13.33 ± 2.16 #‡	6.17 ± 1.17 *	12.83 ± 1.47 #‡	6.50 ± 1.52 *	4.33 ± 1.03 †	12.83 ± 2.23 #‡	5.83 ± 1.47 †
Corticosterone (mg/dL)	1.98 ± 0.08 ‡	2.17 ± 0.46 ‡	2.20 ± 0.31 ‡	1.07 ± 0.05 *#‡§	4.26 ± 0.09 †#	4.25 ± 0.17 †#	2.23 ± 0.18 ‡‖

The results are presented as mean ± standard deviation. For all groups *n* = 6. Symbols (Tukey, *p* < 0.05): * vs. HG (short-term groups: SVOX, SVMS, SVMT); † vs. HG (long-term groups: LVOX, LVMS, LVMT); # vs. SVOX (all other groups compared with SVOX); ‡ vs. LVOX (all other groups compared with LVOX); § SVMT vs. SVOX (metyrapone effect, short-term); ‖ LVMT vs. LVOX (metyrapone effect, long-term). Abbreviations: MDA; malondialdehyde, tGSH; total glutathione, SOD; superoxide dismutase, CAT; catalase, HG; healthy, SVOX; short-term vortioxetine alone, SVMS; short-term vortioxetine + Metyrosine, SVMT; short-term vortioxetine + Methyrapone, LVOX; long-term vortioxetine alone, LVMS; long-term vortioxetine + Metyrosine, LVMT; long-term vortioxetine + Methyrapone.

**Table 3:** Effects of vortioxetine on birth and maternity periods.

Groups	*n*	Non-Infertile Rats		Infertile Rats		Reproductive Process (RP) (Day)	Delay in Maternity (RP-21 Days)
		*n*	%	*n*	%		
HG	6	6	100	0	-	24.5	2.5
SVOX	6	6	100	0	-	24.5	2.5
SVMS	6	6	100	0	-	24.7	2.7
SVMT	6	6	100	0	-	23.3	1.3
LVOX	6	2	33.3	4	66.7	32.5	10.5
LVMS	6	3	50	3	50	33	11
LVMT	6	6	100	0	-	23.7	1.7

Abbreviations: HG; healthy, SVOX; short-term vortioxetine alone, SVMS; short-term vortioxetine + Metyrosine, SVMT; short-term vortioxetine + Methyrapone, LVOX; long-term vortioxetine alone, LVMS; long-term vortioxetine + Metyrosine, LVMT; long-term vortioxetine + Methyrapone. *n* = Number of animals.

## Data Availability

The original contributions presented in this study are included in the article. Further inquiries can be directed to the corresponding author.

## Supplementary Materials

The following supporting information can be downloaded at: https://www.mdpi.com/article/10.3390/ph18111690/s1, Figure S1: Correlation between serum corticosterone and infertility outcome.

## Abbreviations

The following abbreviations are used in this manuscript:

ACTH	Adrenocorticotropic Hormone
ADR	Adrenaline
CAT	Catalase
DOP	Dopamine
EDTA	Ethylenediamine Tetra-Acetic Acid
ELISA	Enzyme-Linked Immunosorbent Assay
GABA	Gamma-Aminobutyric Acid
HPLC	High-Performance Liquid Chromatography
HT	Hydroxytryptamine
MDA	Malondialdehyde
NDR	Noradrenaline
SOD	Superoxide Dismutase
SSRIs	Selective Serotonin Reuptake Inhibitors
tGSH	Total Glutathione
