# Supplementary figures and images for: Effects of Short- and Long-Term Vortioxetine Administration on Reproductive Function in Female Rats
Source: Pharmaceuticals (Basel). 2025 Nov 7;18(11):1690. doi: 10.3390/ph18111690 (PMC12655441; doi:10.3390/ph18111690)

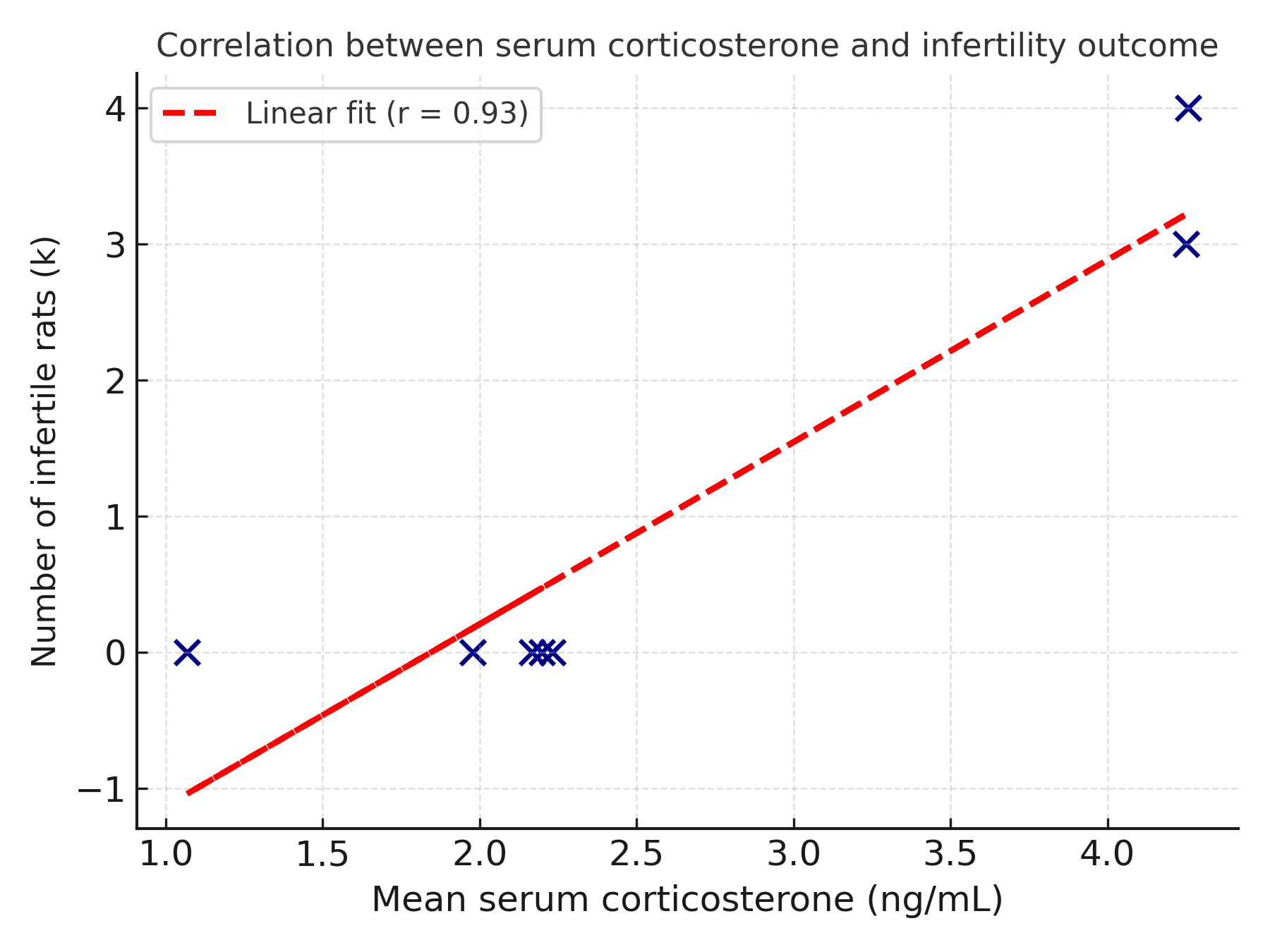

Supplement: Supplementary file 1 [file pharmaceuticals-18-01690-s001.zip › pharmaceuticals-3948448-supplementary.png]
